# Characterization of Three Novel Fatty Acid- and Retinoid-Binding Protein Genes (*Ha-far-1*, *Ha-far-2* and *Hf-far-1*) from the Cereal Cyst Nematodes *Heterodera avenae* and *H*. *filipjevi*

**DOI:** 10.1371/journal.pone.0160003

**Published:** 2016-08-01

**Authors:** Fen Qiao, Lilian Luo, Huan Peng, Shujie Luo, Wenkun Huang, Jiangkuan Cui, Xin Li, Lingan Kong, Daohong Jiang, David J. Chitwood, Deliang Peng

**Affiliations:** 1 The State Key Laboratory for Biology of Insect Pests and Plant Disease, Institute of Plant Protection, Chinese Academy of Agriculture Sciences, Beijing, 100193, P. R. China; 2 College of Plant Science & Technology, Huazhong Agricultural University, Wuhan, 430070, Hubei Province, P. R. China; 3 Nematology Laboratory, USDA-ARS, Building 011A, BARC-West, Beltsville, Maryland, 20705, United States of America; University of Cambridge, UNITED KINGDOM

## Abstract

*Heterodera avenae* and *H*. *filipjevi* are major parasites of wheat, reducing production worldwide. Both are sedentary endoparasitic nematodes, and their development and parasitism depend strongly on nutrients obtained from hosts. Secreted fatty acid- and retinol-binding (FAR) proteins are nematode-specific lipid carrier proteins used for nutrient acquisition as well as suppression of plant defenses. In this study, we obtained three novel FAR genes *Ha-far-1* (KU877266), *Ha-far-2* (KU877267), *Hf-far-1* (KU877268). *Ha-far-1* and *Ha-far-2* were cloned from *H*. *avenae*, encoding proteins of 191 and 280 amino acids with molecular masses about 17 and 30 kDa, respectively and sequence identity of 28%. Protein Blast in NCBI revealed that Ha-FAR-1 sequence is 78% similar to the Gp-FAR-1 protein from *Globodera pallida*, while Ha-FAR-2 is 30% similar to Rs-FAR-1 from *Radopholus similis*. Only one FAR protein Hf-FAR-1was identified in *H*. *filipjevi*; it had 96% sequence identity to Ha-FAR-1. The three proteins are alpha-helix-rich and contain the conserved domain of Gp-FAR-1, but Ha-FAR-2 had a remarkable peptide at the C-terminus which was random-coil-rich. Both Ha-FAR-1 and Hf-FAR-1 had casein kinase II phosphorylation sites, while Ha-FAR-2 had predicted N-glycosylation sites. Phylogenetic analysis showed that the three proteins clustered together, though Ha-FAR-1 and Hf-FAR-1 adjoined each other in a plant-parasitic nematode branch, but Ha-FAR-2 was distinct from the other proteins in the group. Fluorescence-based ligand binding analysis showed the three FAR proteins bound to a fluorescent fatty acid derivative and retinol and with dissociation constants similar to FARs from other species, though Ha-FAR-2 binding ability was weaker than that of the two others. In situ hybridization detected mRNAs of *Ha-far-1* and *Ha-far-2* in the hypodermis. The qRT-PCR results showed that the *Ha-far-1*and *Ha-far-2* were expressed in all developmental stages; *Ha-far-1* expressed 70 times more than *Ha-far-2* in all stages. The highest expression level of *Ha-far-1* was observed in fourth-stage juvenile (J4), whereas the highest expression level of *Ha-far-2* occurred in second-stage juvenile (J2). In conclusion, we have identified two novel *far* genes from *H*. *avenae* and one from *H*. *filipjevi* and have provided further indication that nematode *far* genes are present in a variety of nematode species, where the FAR proteins share similar basic structure, expression pattern and biochemical activities.

## Introduction

Cereal cyst nematodes (CCNs, *Heterodera* spp.) are sedentary plant-parasitic nematodes that infect cereal food crops such as wheat (*Triticum aestivum)*), barley (*Hordeum vulgare*) and oat (*Avena sativa*) [1,2]. CCNs occur in most wheat-growing regions worldwide and cause serious yield losses globally, thereby being major pests affecting the world’s food supply [3,4]. The CCNs are very complex taxonomically and include a group of 12 closely related species, thereby making the control of CCNs much difficult. Among these species, *Heterodera avenae*, *H*. *filipjevi*, and *H*. *latipons* are considered to be most important [4,5]. Unfortunately, wheat production in China is suffering major damage from the first two species, especially *H*. *avenae*, which has spread to Henan, Hebei, and 14 other provinces and cities in less than 30 years since the first report in Hubei Province in China; approximately 20 Mha, or 80% of the total wheat growing regions in China, are infested with *H*. *avenae* and incur yield losses of 20%–30% [2,6–8]. *Heterodera filipjevi* is closely related to *H*. *avenae* in the *H*. *avenae* group complex and was first reported in Henan Province in China [9,10]. This species is also now considered to be an important pest of cereals worldwide, has induced average yield losses of 42% in Turkey and 48% in Iran, and is a great threat to wheat production in China [1,4,11,12].

Like other obligate sedentary endoparasitic nematodes, the life cycles of *H*. *avenae* and *H*. *filipjevi* largely occur within host roots, where ingenious feeding mechanisms have evolved whereby nematodes obtain nutrients from multinucleated syncytia [13,14]. Fatty acids and retinoids are essential compounds that play important roles in cell differentiation, tissue reparation, immune response and the supply of energy [15,16]. Nematodes appear to utilize host-supplied fatty acids and retinoids to sustain their life activities and concurrently impair host lipid-based defenses by manipulating hormone balance [15,17,18]. Because fatty acids and retinoids are hydrophobic and often oxidation sensitive, specific carrier proteins are required for their transport and protection [19–21]. Various classes of lipid carrier proteins have been identified in nematodes, including the nematode polyprotein allergens/antigens (NPAs) and the fatty acid- and retinol-binding (FAR) proteins, which have no counterparts in their host [21–25]. The molecular weights of FAR proteins are about 20kDa, i.e., a little larger than NPAs, whose molecular weights are typically ca. 15kDa [22,26]. Functional analyses indicate that phytoparasitic nematodes secrete FAR proteins with high affinities for fatty acids and retinol and low equilibrium dissociation constants; overexpressing *mj-far-1* in tomato roots lowers the expression of jasmonic acid-responsive genes and increases host susceptibility, thereby promoting the nematode development, reproduction and infection [15,17,27].

The first FAR protein identified and characterized in detail was Ov-FAR-1 (previously known as Ov20) from *Onchocerca volvulus*, which exists in two different molecular mass forms via extent of N-linked glycosylation [21,28]. Subsequently, several FAR proteins were discovered, including ones from other animal-parasitic, plant-parasitic and free-living nematodes [26,29–32]. Unlike several other nematodes, in which only one FAR protein has been found, eight FAR proteins (Ce-FAR-1 to 8) occur in *Caenorhabditis elegans* [31], and two (Ac-FAR-1 and Ac-FAR-2) with 91 percent identity at the amino acid level occur in *Ancylostoma caninum*, which were supposed to possibly be the first reported *far* alleles in a nematode [26].

In the destructive CCNs *H*. *avenae* and *H*. *filipjevi*, study on FAR proteins is still in its infancy. The aim of this investigation was characterize the FAR genes from *H*. *avenae* and *H*. *filipjevi*. The objectives of our study included obtaining the full length cDNA of FAR genes from *H*. *avenae* and *H*. *filipjevi* by RT-PCR and RACE technology, detecting the mRNA with *in situ* hybridization and analyzing transcriptional levels using qRT-PCR, and measuring the binding activities with fluorescence-based assays.

## Materials and Methods

### Nematode Populations

The cysts of *Heterodera avenae* and *H*. *filipjevi* were isolated from wheat roots of Daxing district, Beijing, and Xuchang County of Henan province, China in 2010. Nematode species of *H*. *avenae* and *H*. *filipjevi* were clearly distinguished by species-specific PCR fragments with 242 and 170bp of ITS sequences, respectively [33]. Cereal cyst nematodes *H*. *avenae* and *H filipjevi* were maintained on a compatible wheat *Triticum aestivum* cv. Wenmai 19 at 16°C in a greenhouse for the first week and then 22°C for the remainder of the 6 weeks growth period, mature cysts were collected, and Cysts were incubated at 4°C for at least 8 weeks, and then transferred to 16°C for hatching, preparasitic second-stage juveniles (pre-J2) were hatched as previously described [34]. The freshly hatched pre-J2 were collected on 25μm aperture sieves, suspended in DEPC-treated water, counted under a microscope, and used for inoculation and DNA or RNA extraction.

### DNA and RNA Extraction

Genomic DNA of *H*. *avenae* was extracted from mature cysts as described previously [35]. Total RNA of *H*. *avenae* and *H*. *filipjevi* were isolated from about 100,000 pre-J2s with TRIzol reagent (Invitrogen, USA) according to the manufacturer’s instructions and then treated with RQ1 RNase-Free DNase (Promega, USA) to remove the genomic DNA. The concentration and quality of RNA was determined with a NanoDrop-1000 (Thermo Fisher Scientific Inc., Waltham, USA) and 1% agarose gel. cDNA was synthesized from 1μg total RNA with Oligo(dT)_18_ primers by using SuperScript™ III First-Strand Synthesis kit (Invitrogen, USA) according to the manufacturer’s instructions.

### Cloning of Full Length FAR Genes from *H*. *avenae* and *H*. *filipjevi*

Two candidate FAR genes (termed *Ha-far-1* and *Ha-far-2*) and one candidate FAR gene (named *Hf-far-1*) were identified from our transcriptome sequence data of *H*. *avenae* and *H*. *filipjevi* (unpublished), respectively. Accordingly, specific primers of *Ha-far-1* (Ha-far-1F and Ha-far-1R), *Ha-far-2* (Ha-far-2F and Ha-far-2R) and *Hf-far-1* (Hf-far-1F and Hf-far-1R) (Table 1) were designed to amplify the cDNAs encompassing complete predicted open reading frames (ORFs). A RACE method described by the manufacturer’s instructions in the GeneRacer™ kit (Invitrogen, USA) was adopted to confirm the 5’cDNA end and the 3’cDNA end of *Ha-far-2 and Hf-far-1*. In this procedure, specific 5’RACE primers (5’Ha-far-2 and 5’nested Ha-far-2), 3’RACE primers (3’Ha-far-2 and 3’nested Ha-far-2) (Table 1) and the anchor primers supplied in the GeneRacer™ kit were used for *Ha-far-2* cloning. The 3’end cDNA of *Hf-far-1* was confirmed in the same way with specific 3’ RACE primers (3’Hf-far-1 and 3’nested Hf-far-1) (Table 1). PCR products were purified and cloned into pGEM-T Easy Vector (Promega, USA) and sequenced. Finally, fragments were spliced into the complete *Ha-far-1* and *Hf-far-1* sequences. *Ha-far-1* is full length from transcriptome sequence data with a polyA tail. gDNA of *Ha-far-1* and *Ha-far-2* were amplified from extracted genomic DNA of *H*. *avenae* with gHa-far-1F and gHa-far-1R, gHa-far-2F and gHa-far-2R (Table 1), respectively.

**Table 1:** Primers used in this study.

Primer name	Primer sequence (5’- 3’)
Ha-far-1F	ACAAACACCCGCCAACCAT
Ha-far-1R	AAGTGCCACGGCGTTTAGG
Ha-far-2F	CCCCCCTTGATAATGCTAATAAT
Ha-far-2R	CATTGCTGTTGCTACCGTTGAT
Hf-far-1F	ATAAACACCCGCCAACCATC
Hf-far-1R	TCCATCAATCCGTCCGTTCT
5’Ha-far-2	CTGATAAGTGTCCGCTTTAGCCAAC
5’nested Ha-far-2	CTGATAAGTGTCCGCTTTAGCCAAC
3’Ha-far-2	ATGGGAAGGGAAGGTGGAGGGATG
3’nested Ha-far-2	GAACCAATCAACGGTAGCAACAGCA
3’Hf-far-1	TTACAACACACTGACCGAAGAGGAC
3’nested Hf-far-1	CGAAGGCGGGAGAGAAACCGAACC
GeneRacer™5′Primer	CGACTGGAGCACGAGGACACTGA
GeneRacer™5′Nested Primer	GGACACTGACATGGACTGAAGGAGTA
GeneRacer™3′Primer	GCTGTCAACGATACGCTACGTAACG
GeneRacer™3′Nested Primer	CGCTACGTAACGGCATGACAGTG
gHa-far-1F	ATGAATGCCGTTCTCTCCTCTC
g Ha-far-1R	TTAGGCGGCTGGTGCGGCAC
gHa-far-2F	ATGCTAATAATGATTATGCCTCTGC
g Ha-far-2R	CTACCGTTGATTGGTTCTTCCGT
Ha-far-1-NcoI	CATGCCATGGGGGCCACTTTGCCACCCATTGA
Ha-far-1-XhoI	CTGCTCGAGTTAGGCGGCTGGTGCGGCAC
Ha-far-2-NcoI	CATGCCATGGGGTTCCCCGCCGTTTTGACAACA
Ha-far-2-XhoI	CTGCTCGAGCTACCGTTGATTGGTTCTTCC
Hf-far-1-NcoI	CATGCCATGGGGGCCACTTTGCCACCCATTGA
Hf-far-1-XhoI	CTGCTCGAGTTAGGCGGCCGGTGCAGCAC
iHa-far-1F	CACACTGACCGAGGAGGACAA
iHa-far-1R	GCTCCTCCAGATTCGGCTT
iHa-far-2F	ACGGTTGCCTTCGTTTTGG
iHa-far-2R	ATCGTCCATCCCTCCACCTT
ActinF	ACTGTTCCAGCCATCCTTCATC
ActinR	GACAGCACGTTGTTGGCGTAC
GAPDH-qS1	AGCGGCACAGAACATCATCC
GAPDH-qAS1	GGTCCTCCGTGTAGCCCAAA
qHa-far-1F	ACCTGGTCAAGGGGAAAATTG
qHa-far-1R	TGCGAAGCTCCTCCAGATTC
qHa-far-2F	AAGCAGTCCAAGTGGTCACCTC
qHa-far-2R	CCTTCAAATTGGAGAGCGAAAC

### Sequence Analysis, Alignment and Phylogenetics

The sequence homology searches to non-redundant protein database (nr) and non-redundant nucleotide database (nt) was performed via the BLASTx and BLASTn programs at NCBI (http://blast.ncbi.nlm.nih.gov/Blast.cgi, 2015). Coding sequences (CDSs) of *Ha-far-1*, *Ha-far-2* and *Hf-far-1* were predicted by NCBI ORF Finder (http://www.ncbi.nlm.nih.gov/gorf/gorf.html). Conserved domains were analyzed by RPS-BLAST in NCBI. Signal peptides were predicted through SignalP 4.1 Server (http://www.cbs.dtu.dk/services/SignalP/) and PSORT II Prediction (http://psort.hgc.jp/form2.html). The molecular weight, theoretical pI and formula were computed using ProtParam tool (http://web.expasy.org/protparam/); NetNGlyc 1.0 Server (http://www.cbs.dtu.dk/services/NetNGlyc/) and KinasePhos (http://kinasephos.mbc.nctu.edu.tw/) were used to predict the N-glycosylation sites and casein kinase II phosphorylation sites, respectively. Protein secondary structure predictions were performed at PBIL (https://npsa-prabi.ibcp.fr/cgi-bin/npsa_automat.pl?page=/NPSA/npsa_hnn.html). Multiple protein sequence alignments and phylogenetic tree analyses were performed with Molecular Evolutionary Genetics Analysis version 6.0 (MEGA6.0) [36] by the neighbor-joining statistical method after aligning the protein sequences with ClustalW.

### Expression and Purification of Ha-far-1, Ha-far-2 and Hf-far-1 Recombinant Proteins

PCR primers incorporated with NcoI or XhoI restriction sites of *Ha-far-1* (Ha-far-1-NcoI and Ha-far-1-XhoI), *Ha-far-2* (Ha-far-2-NcoI and Ha-far-2-XhoI) and *Hf-far-1* (Hf-far-1-NcoI and Hf-far-1- XhoI) (Table 1) were used to obtain full-length cDNA exclusive of the putative signal peptides, then the amplification products were ligated to pGEM-T easy vector for sequence confirmation. The correct sequences were subcloned into pHAT2-His-tagged expression vector (kindly supplied by professor Jun-Feng Liu). Recombinant pHAT2 plasmids were introduced into *E*. *coli* BL21 (DE3) cells (Novagen, Germany). Recombinant proteins induced with 1 mM isopropyl β-D-thiogalactopyranoside (IPTG) were purified with Ni Sepharose High Performance (GE Healthcare, Sweden), and imidazole was removed with dialysis. Sodium dodecyl sulfate polyacrylamide gel electrophoresis (SDS-PAGE) was used to detect the purified recombinant proteins.

### Spectrofluorimetry and Ligand Binding Assays

The binding activity of purified Ha- FAR-1, Ha- FAR-*2* and Hf-FAR-1 proteins to fatty acid was measured using 11-((5-dimethylaminonaphthalene-1-sulfonyl) amino) undecanoic acid (DAUDA) (Sigma, USA) and retinol (Sigma, USA). In competition experiments, oleic acid (Sigma, USA) was used as a competitor of DAUDA or retinol. The monochromatic excitation wave lengths used for DAUDA and retinol were 345 nm and 350 nm, respectively. The equilibrium dissociation constant (K_d_) was estimated by fluorescence titration [14, 25, 28]. The concentrations of protein solutions were gradually increased in 3 mL of PBS containing 3 μM DAUDA to determine the Kd of Ha-FAR-1 and Hf-FAR-1 binding to DAUDA, and containing 15 μM DAUDA for Ha-FAR-2 binding to DAUDA. The K_d_ for Ha-FAR-1, Ha-FAR-2 and Hf-FAR-1 binding to all-trans-retinol was estimated by adding increasing concentrations of all-trans-retinol to 5 μM protein in PBS.

### In Situ Hybridization

Gene-specific primers (iHa-far-1F, iHa-far-1R and iHa-far-2F, iHa-far-2R) (Table 1) were designed to locate the mRNA of Ha-far-1 and Ha-far-2 by performing in situ hybridization as described previously [36]. PCR DIG Probe Synthesis Kit (Roche, Switzerland) was used to synthesize digoxigenin (DIG)-labeled sense and antisense cDNA, and DIG High Primer and Detection Starter Kit I (Roche, Switzerland) was used for hybridization following the manufacturer’s instructions.

### Expression Pattern of *Ha-far-1* mRNA at Different Development Stages of *H*. *avenae*

Quantitative real-time reverse transcription-PCR (qRT-PCR) was performed to analyze the expression pattern of *Ha-far-1* (Primers: qHa-far-1F and qHa-far-1R) and *Ha-far-2* (Primers: qHa-far-2F and qHa-far-2R) (Table 1) among different developmental stages. The β-actin gene (Primers: ActinF and ActinR) and glyceraldehyde 3 phosphate dehydrogenase gene (Primers: GAPDH-qS1 and GAPDH- qAS1) [37] (Table 1) were used as a reference gene. One or both of the specific primer pairs crossed two exons. Total RNA was extracted from pre-parasitic J2s, J3s, J4s and mature females of *H*. *avenae*, and parasitic J2s were from wheat root infested by *H*. *avenae* at 1dpi (day past inoculation). After removal of contaminating genomic DNA by RQ1 RNase-Free DNase (Promega, USA), 1μg total RNA was reverse transcripted into cDNA. The qRT-PCR was carried out with triplicate technical replicas by SYBR qPCR SuperMix-UDG w/ROX (Invitrogen Corporation, Carlsbad, CA, USA) on an ABI 7500 Fast RT-PCR System (Applied Biosystems Inc., USA). The data were analyzed by the ∆∆Ct method and standardized to the β-actin gene expression levels.

## Results

### Three Full-Length FAR Genes from *H*. *avenae* and *H*. *filipjevi*

**Fig 1:**
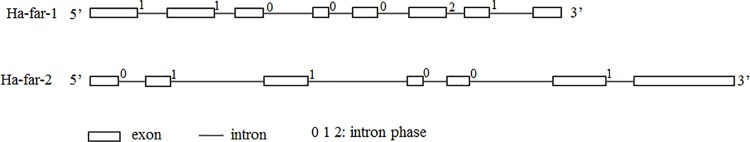
Structure of *Heterodera avenae* FAR protein genes *Ha-far-1* and *Ha-far-2*. The structures of *Ha-far-1* and *Ha-far-2* were determined by genomic DNA to cDNAs from start codon to stop codon. Exon (rectangles), introns (solid lines) and intron phase (0, 1, 2) were shown.

Based on the ESTs obtained from a transcriptome library (unpublished), we identified two fragments from *H*. *avenae* and one from *H*. *filipjevi* which displayed high sequence and structural similarity to other known FARs and were termed *Ha-far-1*, *Ha-far-2* and *Hf-far-1* for the genes and Ha-FAR-1, Ha-FAR-2 and Hf-FAR-1 for proteins and the GenBank accession numbers are KU877266, KU877267 and KU877268 respectively. The full length cDNA sequence of *Ha-far-1* in the transcriptome was 820bp with a 23bp polyA tail. RACE technology was used to amplify the 5’ and 3’ ends of *Ha-far-2* and 3’ end of *Hf-far-1*. After alignment and splicing, cDNA sequences containing complete ORFs of *Ha-far-1*, *Ha-far-2* and *Hf-far-1* were obtained of 820 bp, 1160 bp and 801 bp, respectively, and according to the NCBI ORF finder, the ORFs were 576 bp, 843 bp and 576 bp, which encode 191, 280 and 191 amino acids, respectively. Based on the cDNA sequences, additional primers were designed to amplify the gDNA of *Ha-far-1* and *Ha-far-2* which were 1212 bp from ATG to TAA, contained 7 introns and 8 exons for *Ha-far-1* (Fig 1) and 2174bp from ATG to TAG, and contained 6 introns and 7 exons for *Ha-far-2* (Fig 1).

### Sequence Analysis of the Ha-FAR-1, Ha-FAR-2 and Hf-FAR-1 Proteins

**Fig 2:**
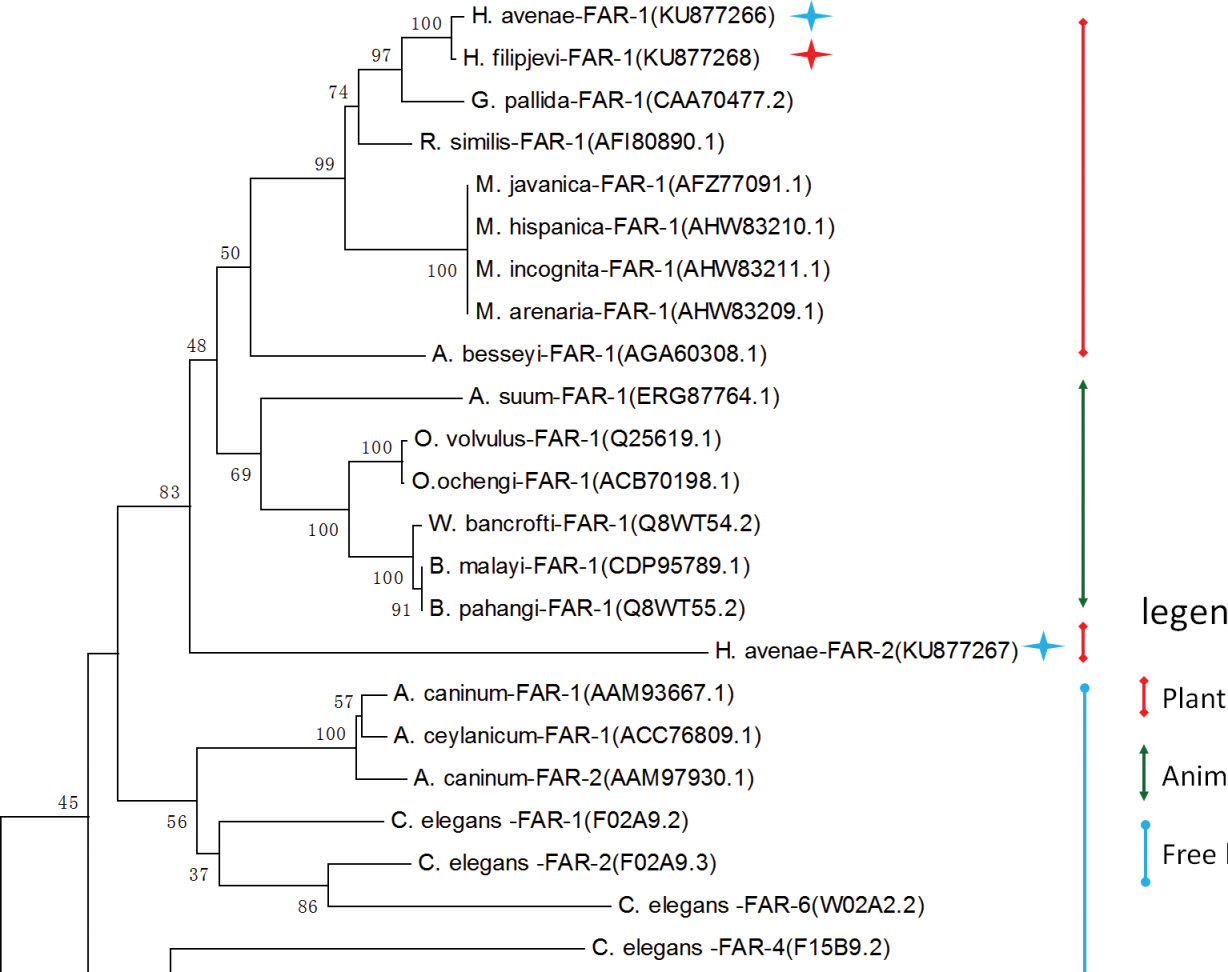
Phylogenetic tree containing FAR proteins of nematodes. A neighbor-joining phylogenetic tree with protein sequences of Ha-FAR-1, Ha-FAR-2, Hf-FAR-1 (marked with asterisks) and the 24 amino acid sequences of FAR proteins from free-living, animal-parasitic and plant-parasitic nematodes. The phylogenetic tree was constructed by Mega 6.0 after aligning the protein sequences with ClustalW. The percentage bootstrap values were inferred from 1,000 replicates and are indicated at the nodes. Scale bar represents 0.2 amino acid substitutions per site.

**Fig 3:**
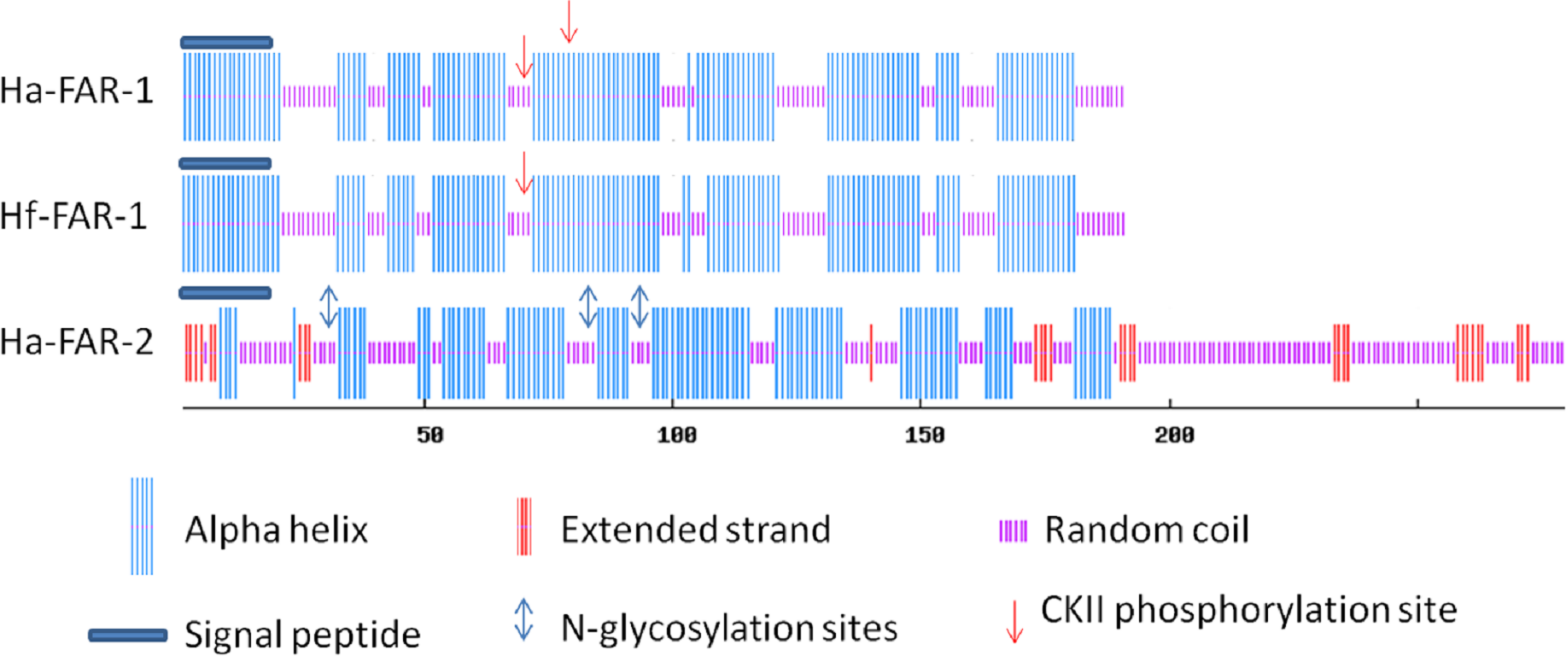
Secondary structure of cereal cyst nematode FAR proteins. Ha-FAR-1,Ha-FAR-2 and Hf-FAR-1 contained a putative signal peptide at the N terminus (long dark blue rod) and were rich in alpha helix (long vertical blue lines) and random coil (short vertical violet lines) structures. An extended strand (moderate vertical red lines) was detected in Ha-FAR-2. Ha-FAR-1 had two predicted casein kinase II phosphorylation sites at amino acids 71 and 81, and Hf-FAR-1 has one at amino acid 71 (red arrow). NetNGlyc 1.0 Server indicated that neither Ha-FAR-1 nor Hf-FAR-1 has a predicted N-glycosylation site but that Ha-FAR-2 contains predicted N-glycosylation sites (double sided blue arrows) at amino acids 32, 84 and 94.

The predicted Ha-FAR-1 and Hf-FAR-1 proteins both contained 191 amino acids and had calculated molecular mass of 21157.3 Da and 21173.3 Da and molecular formula of C_946_H_1539_N_247_O_291_S_4_ and C_950_H_1539_N_247_O_289_S_4_ and theoretical pI of 5.62 and 5.63, respectively. Protein Blast in NCBI revealed that both Ha-FAR-1 and Hf -FAR-1 were most similar to the Gp-FAR-1 protein from *Globodera pallida* (GenBank accession number CAA70477) with 78% (E-value 2e-90) and 77% (E-value 2e-90) protein identity respectively and 30–73% identity to other FAR proteins. Alignment of Hf-FAR-1 and Ha-FAR-1 by bl2seq showed that they were highly homologous with only 7 different amino acids (96% identity, 0 gap, E-value 2e-135). Interestingly, Ha-FAR-2, which consisted of 280 amino acids, significantly differed from the other two proteins. The theoretical molecular mass was 31219.2 Da with a molecular formula of C_1360_H_2200_N_404_O_426_S_6_ and a theoretical pI of 9.02. The Blast analysis showed that Ha-FAR-2 had the highest similarity to a FAR protein from *Radopholus similis* (GenBank accession number AFI80890.1, 31% identity and E-value 2e-21), and the identity between Ha-FAR-1 and Ha-FAR-2 was 28% (E-value 9e-25). The phylogenetic tree was constructed with amino acid sequences of Ha-FAR-1 (KU877266), Ha-FAR-2 (KU877267), Hf-FAR-1 (KU877268) and other 24 FAR proteins from 16 species including 8 animal-parasitic and 7 plant-parasitic nematodes and the free-living nematode *C*. *elegans* (Fig 2). The tree indicated that *Ha*-FAR-1, *Ha*-FAR-2 and *Hf*-FAR-1were present in one group constructed of 13 FAR proteins from plant and animal-parasitic nematodes. *Ha*-FAR-1 and *Hf*-FAR-1 adjoined each other in the plant-parasitic nematode branch, but *Ha*-FAR-2 seemed to be self-contained within the plant-parasitic nematode and the animal-parasitic nematode clusters (Fig 2). SignalP and PSORT II Prediction program analyses showed that *Ha*-FAR-1, *Ha*-FAR-2 and *Hf*-FAR-1 possessed 21, 23 and 21 aa cleavable hydrophobic secretary signal peptides at the N terminus, respectively, suggesting that these three proteins are secreted like other FARs (Fig 3). Protein secondary structures predictions performed at PBIL showed that the three proteins were alpha-helix-rich and no beta-sheet was detected, but Ha-FAR-2 had a remarkable peptide at the C-terminus which was random-coil-rich (Fig 3). Furthermore, sequence analysis has showed that Ha-FAR-1, Ha-FAR-2 and Hf-FAR-1 all contained a conserved domain of Gp-FAR-1, spanning from amino acids 31–182 (E-value 4.04e-58), 49–190 (E-value 1.14e-24), and 31–182 (E-value 1.73e-57), respectively, indicating that they belong to the fatty acid- and retinoid-binding (FAR) family of proteins (Fig 3). Both Ha-FAR-1 and Hf-FAR-1 had casein kinase II phosphorylation sites but no N-glycosylation site (Fig 3). In contrast, Ha-FAR-2 was predicted to have N-glycosylation sites, but no casein kinase II phosphorylation site (Fig 3).

### Ligand Binding

**Fig 4:**
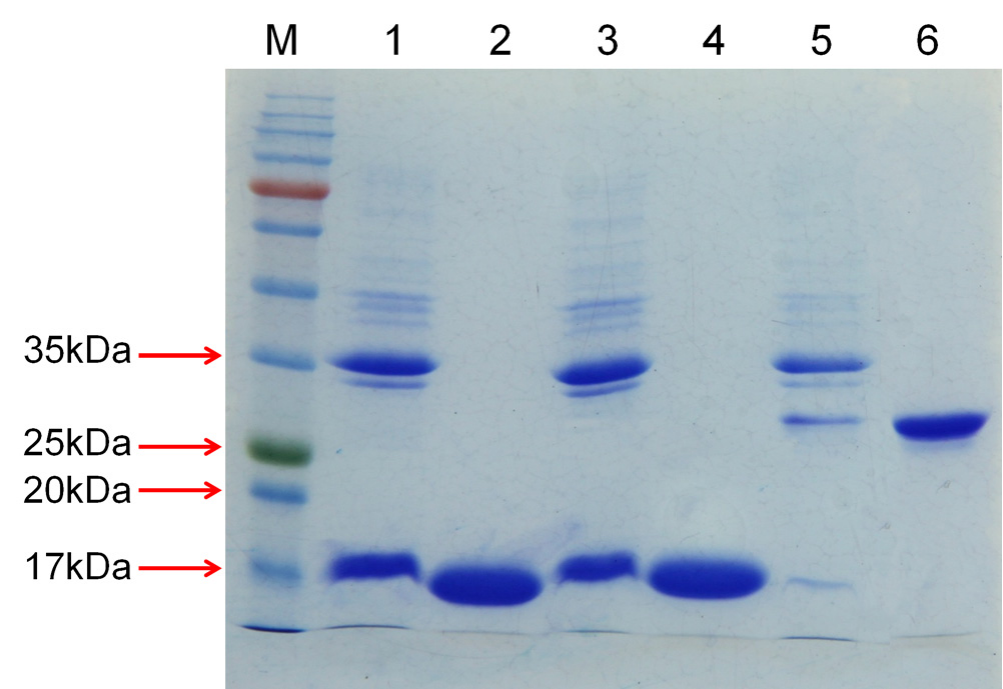
Detection of recombinant proteins of Ha-FAR-1, Ha-FAR-2 and Hf-FAR-1 via SDS-PAGE M: Protein marker; 1, 3, 5: Supernatant of unpurified Hf-FAR-1, Ha-FAR-1, Ha-FAR-2 respectively; 2, 4, 6: Purified Hf-FAR-1, Ha-FAR-1, Ha-FAR-2 respectively.

**Fig 5:**
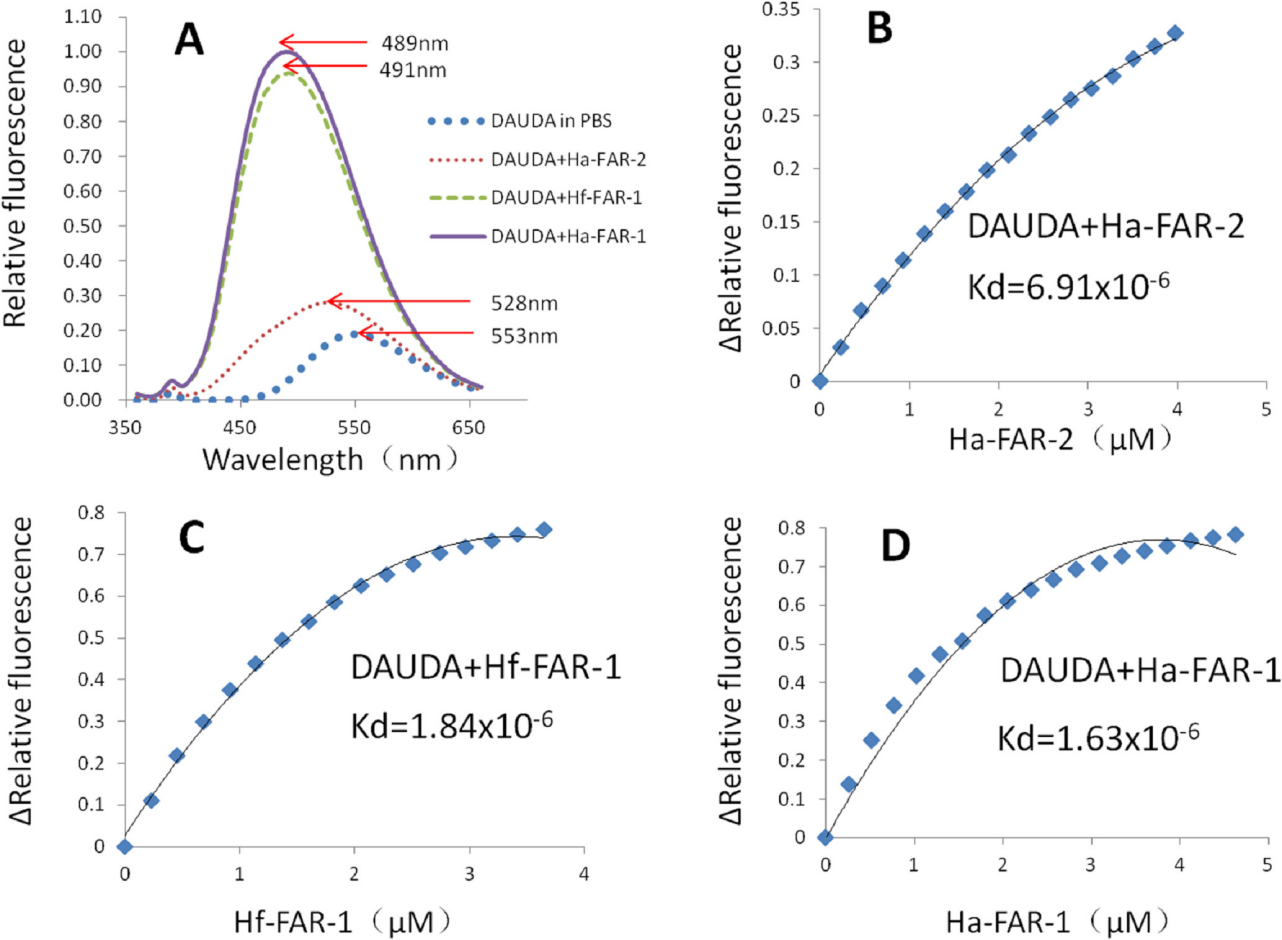
Diverse binding activates to DAUDA of Ha-FAR-1,Ha-FAR-2 and Hf-FAR-1. A: Fluorescence emission spectra (excitation at 345 nm) of 3μM DAUDA in buffer alone or with buffer plus, 3μM Ha-FAR-1 complex, 3μM Ha-FAR-2 complex or 3μM Hf-FAR-1 complex. B, C, D: Titration curves for determining the dissociation constant (Kd) for interaction of DAUDA with Ha-FAR-2, Hf-FAR-1 and Ha-FAR-1.

**Fig 6:**
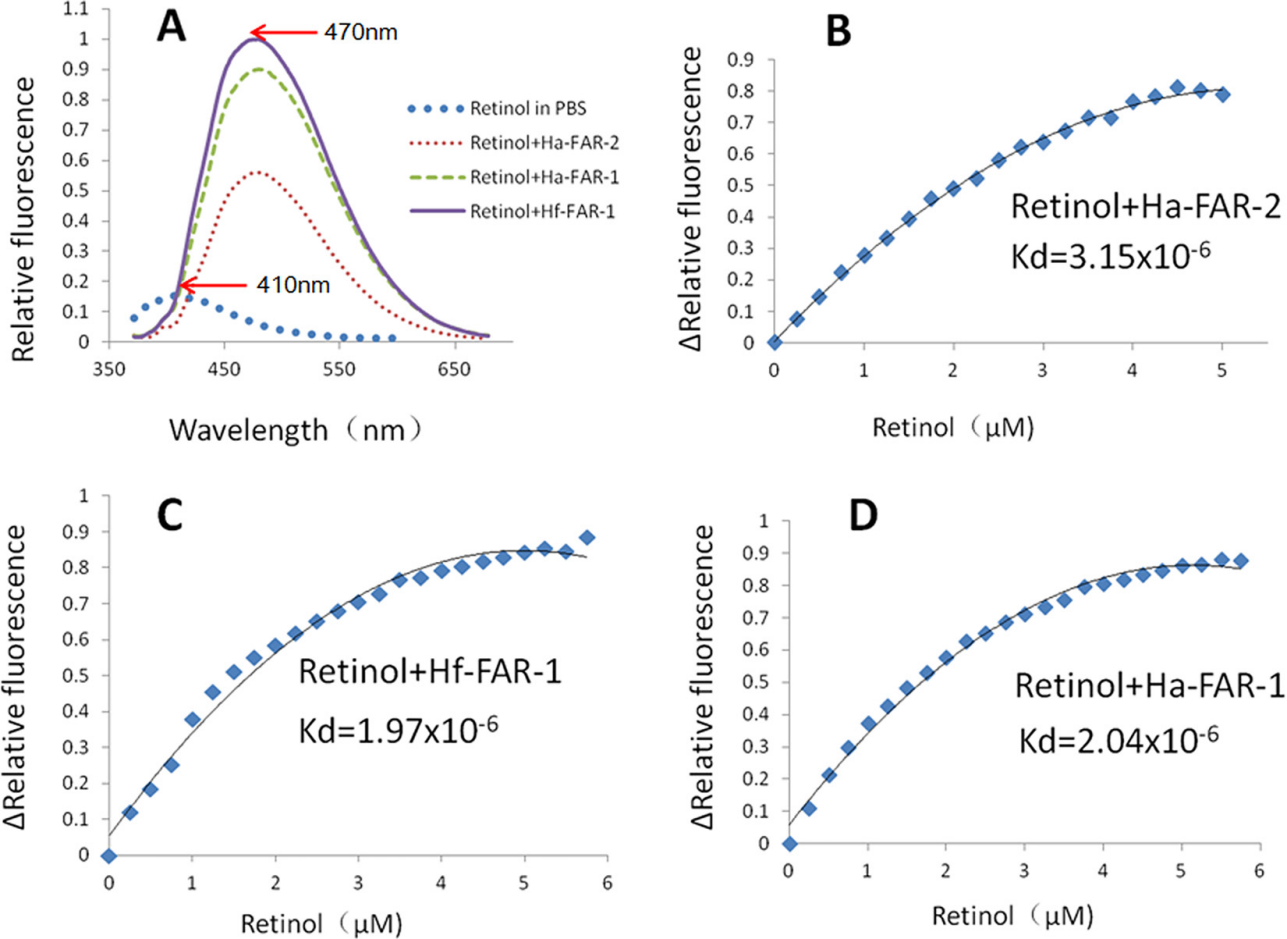
Diverse binding abilities to retinol of Ha-FAR-1,Ha-FAR-2 and Hf-FAR-1. A: Fluorescence emission spectra (excitation at 350 nm) of 10μM retinol in buffer alone or in buffer plus 2.5μM Ha-FAR-1 complex, 2.5μM Ha-FAR-2 complex or 2.5μM Hf-FAR-1 complex. Peak emission was at 470nm. B, C, D: Titration curves for determining the dissociation constant (Kd) for interaction of retinol with Ha-FAR-2, Hf-FAR-1 and Ha-FAR-1.

The His fusion recombinant proteins of Ha-FAR-1, Ha-FAR-2 and Hf-FAR-1 were expressed in *E*. *coli* BL21 (DE3) cells. SDS-PAGE analysis showed the proteins existed in the supernatant, thus indicating that they were soluble proteins (Fig 4: lane 1, 3, 5). After purification, only single bands for Ha-FAR-1, Hf-FAR-1 and Ha-FAR-2 were observed and were approximately 17 kDa,17 kDa and 30 kDa, respectively (Fig 4: lane 2, 4, 6), indicating that the purification was effective. All three purified proteins had binding activities to DAUDA with blue shifts in their peak emission, but degrees were quite diverse. For absence and presence of Ha-FAR-1, the peak fluorescence emission shifted from 553 nm to 489 nm, and for the presence of Hf-FAR-1, from 553 nm to 491 nm, but for the presence of Ha-FAR-2 only to 528 nm, thus indicating that Ha-FAR-1and Hf-FAR-1 had similar polar binding sites but that the polar binding site of Ha-FAR-2 was much weaker (Fig 5A). Fluorescence emission intensity was increased after addition of the purified proteins into solutions of retinol, indicating that retinol had been removed from the solvent buffer by protein polar binding sites (Fig 6A). Similarly, the much larger shifts induced by Ha-FAR-1 and Hf-FAR-1 than Ha-FAR-2 indicate that, as with retinol, there were more active binding sites for DAUDA din Ha-FAR-1 and Hf-FAR-1 than Ha-FAR-2.

**Fig 7:**
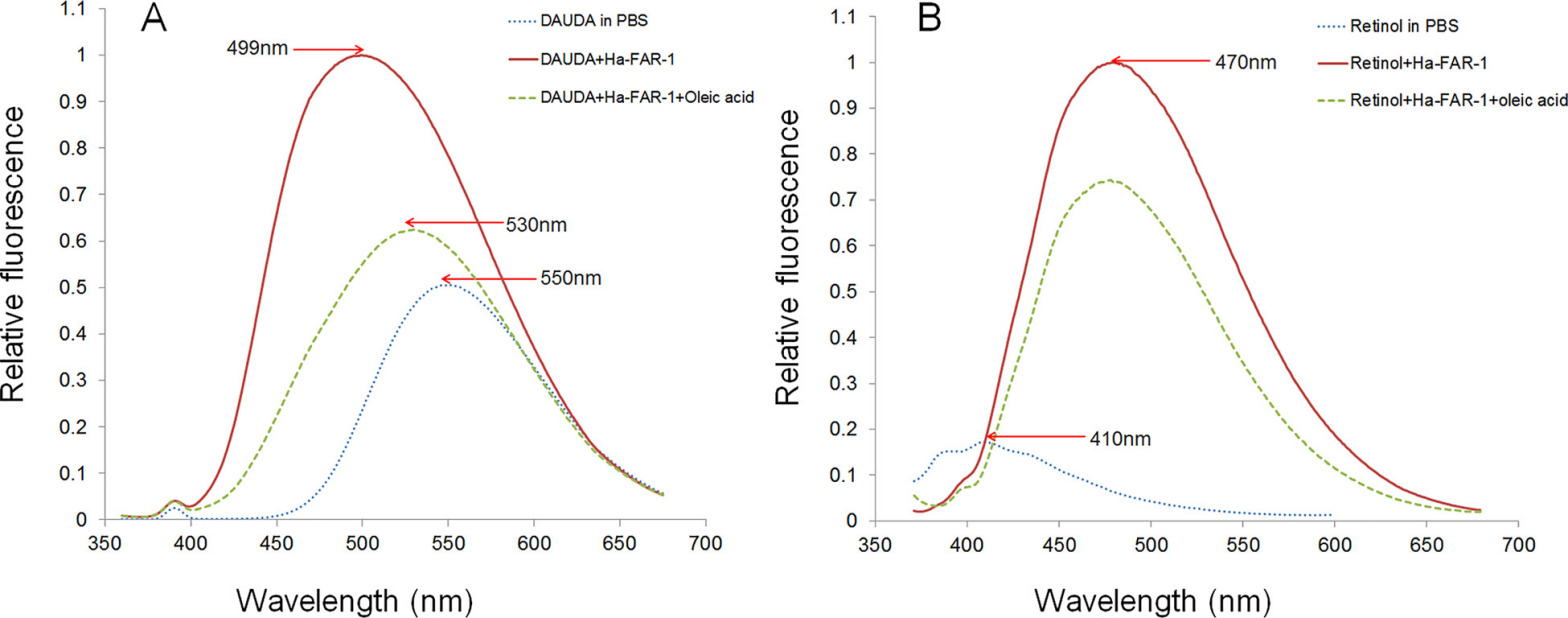
**Competition analysis of oleic acid-displaced DAUDA (A) or retinol (B) from Ha-FAR-1 binding sites.** A: The reversed change of fluorescence intensity (excitation at 345 nm) was observed after the addition of oleic acid to the 10μM DAUDA +1.5μM Ha-FAR-1 complex. The wavelengths of peak emission by DAUDA were changed from 499 nm to 530 nm. B: Fluorescence intensity (excitation at 350 nm) produced a pronounced drop after adding oleic acid to 10μM retinol +2.5μM Hf-FAR-1 complex.

The binding affinities of FAR proteins to DAUDA and retinol were further measured by vitro titration analysis. The in vitro equilibrium dissociation constants (Kds) for interaction with DAUDA by Ha-FAR-1, Hf-FAR-1 and Ha-FAR-2 were 1.63x10^-6^, 1.84x10^-6^ and 6.91x10^-6^, respectively (Fig 5B–5D). The values for interaction with retinol by Ha-FAR-2, Hf-FAR-1 and Ha-FAR-1 were 3.15x10^-6^, 1.97x10^-6^, and 2.04x10^-6^, respectively (6B-D) which were all within micromolar ranges as other reported FARs [15]; however, binding ability of Ha-FAR-2 to DAUDA and retinol was significantly poorer than Ha-FAR-1and Hf-FAR-1. Competition experiments showed that fluorescence intensity of DAUDA or retinol produced a pronounced drop after adding oleic acid, and for DAUDA, red shifts in their peak emission from 499nm to 530nm were detected (Fig 7), indicating that DAUDA and retinol have the same or interactive binding sites and can be competitively displaced by oleic acid.

### Expression and Localization of *Ha-far-1* mRNA

**Fig 8:**
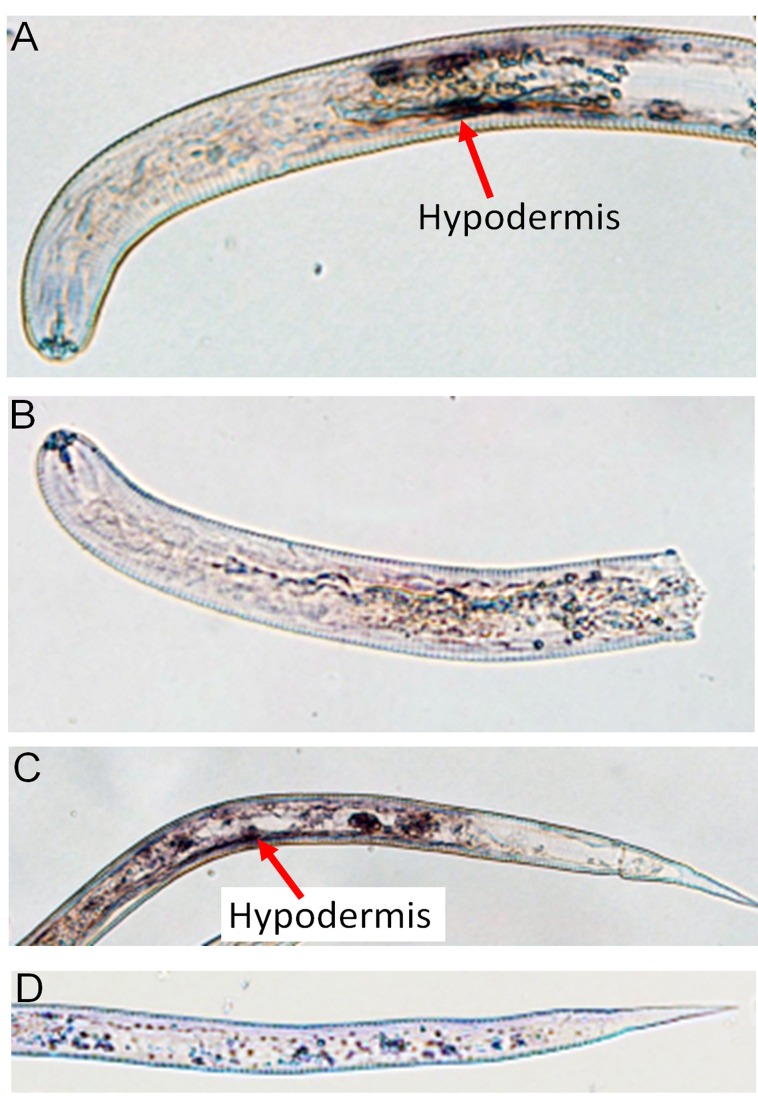
Localization of *Ha-far-1* and *Ha-far-2* in pre-J2 of *H*. *avenae by* in situ hybridization. Hybridization to antisense *Ha-far-1* (A) or *Ha-far-2* (C) by a DIG-labeled cDNA probe showed that the *Ha-far-1*and *Ha-far-2* are both located in the hypodermis. The hybridization with DIG- labeled sense cDNA probe of *Ha-far-1* (B) or *Ha-far-2* (D) was used as control.

**Fig 9:**
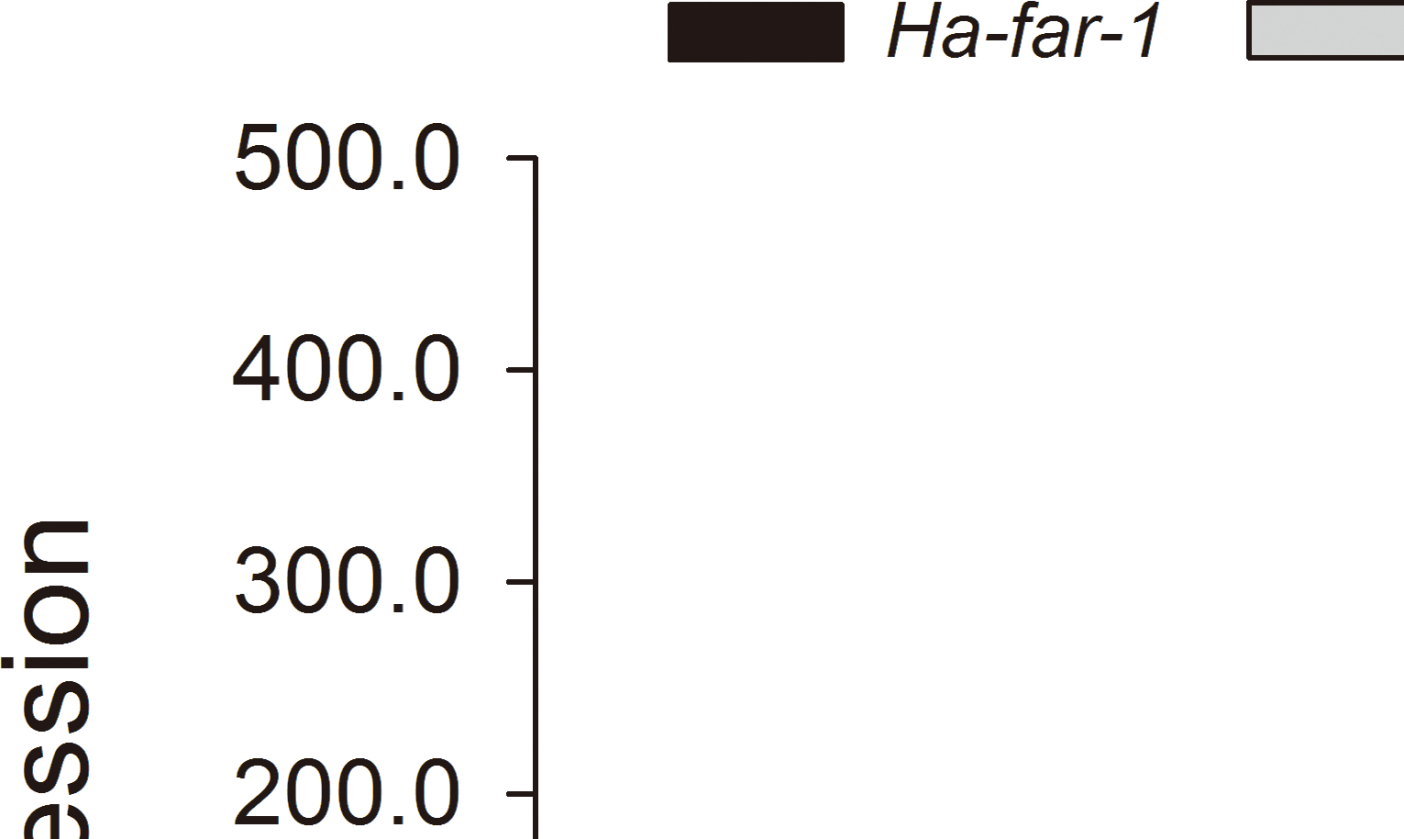
Relative expression of *Ha-far-1* and *Ha-far-2* in five developmental stages was determined using RT-qPCR. The relative expression was calculated with 2^-∆∆Ct^ method by normalizing with two internal reference β-actin gene and GAPDH gene and presented as the change in mRNA level in various nematode developmental stages relative to that of *Ha-far-2 at* preJ2. preJ2: pre-parasitic second-stage juvenile; J2, J3 and J4: second-, third- and fourth-stage juvenile, respectively; F: female.

The mRNA of *Ha-far-1* and *Ha-far-2* were detected by in situ hybridization in pre-parasitic juveniles of *H*. *avenae*. The result showed that the DIG-labeled antisense probes of *Ha-far-1*and *Ha-far-2* both hybridized in the hypodermis (Fig 8A and 8C). No signal in control groups was detected by hybridization with the DIG-labeled sense probes (Fig 8B and 8D). The qRT-PCR results showed that *Ha-far-1*and *Ha-far-2* were expressed in all developmental stages, while the expression level of *Ha-far-1* was significantly greater than that of *Ha-far-2* in all developmental stages (Fig 9). The highest expression level of *Ha-far-1* was observed in J4, whereas for *Ha-far-2* the highest expression level was observed in J2 (Fig 9).

## Discussion

FAR proteins are unique lipid carrier proteins in nematodes and are essential for the infection process and for completing the nematode life cycle [29,32]. Retinoids appear to be one class of molecules of importance to nematodes; for example. host nodules induced by the river blindness nematode *Onchocerca volvulus* contained eight times more retinol than the surrounding skin [38].With respect to plant-parasitic nematodes, much less was known about FAR proteins and lipid synthesis in plant nematodes than in animal parasites. But overexpression of mj-far-1 in tomato roots regulated plant cell wall-, hormone- and fatty acid-related genes and suppressed the expression of jasmonic acid-responsive genes, thereby increasing host susceptibility and promoting nematode development, reproduction and infection [16,17]. Whether plant- or animal-parasitic, nematodes may acquire fatty acids and retinoids to meet their developmental and metabolic needs and to disturb host physiology. Consequently, the investigation of FAR proteins is important for understanding host response and nematode pathogenesis.

In this study, we identified two novel functional FAR proteins (Ha-FAR-1 and Ha-FAR-2) from *H*. *avenae* and one FAR protein (Hf-FAR-1) from *H*. *filipjevi*. The primary and secondary structures of these proteins shared much in common with FAR proteins secreted by other nematodes (Fig 3) which are alpha-helix-rich and contain a conserved FAR domain indicating that they belong to the fatty acid- and retinoid-binding (FAR) family of proteins [31,32]; Ha-FAR-2 had a long peptide at the C terminus which was random-coil rich (Fig 3). Both Ha-FAR-1 and Hf-FAR-1 apparently have casein kinase II (CKII) phosphorylation sites but no N-glycosylation site, which is conserved in many FAR proteins [26,27]. In the eight *C*. *elegans* FAR proteins (Ce-FAR-1 to -8), the CKII phosphorylation site is conserved within the family and the ligand binding activity of Ce-FAR-7 increases after phosphorylation by this kinase [39]. Interestingly, the Ha-FAR-2 sequence predicts it has N-glycosylation sites but no CKII phosphorylation site (Fig 3).

The FAR proteins from *O*. *volvulus* occur in two isoforms with different molecular masses of 20 and 22 kDa, which results from different levels of N-linked glycosylation rather than sequence variation [40]. A study of FAR proteins from filarial nematodes showed that FAR proteins are differentially regulated by post-translational modification but that biochemical activities are strongly conserved [41]. It is possible that Ha-FAR-1 and Ha-FAR-2 are also modified post-translationally differently. Our ligand binding assays showed that Ha-FAR-1, Ha-FAR-2 and Hf-FAR-1 exhibited significant binding activities to fatty acids and retinol (Figs 5 and 6), thereby indicating that these three proteins might be involved in sequestering lipids from the nematode host. Similarly, FAR proteins from *Aphelenchoides besseyi* (Ab-FAR-1), *Radopholus similis* (Rs-FAR-1) and *Ancylostoma caninum* (Ac-FAR-1) have high affinity for fatty acids and retinol [15,25,26]. Interestingly, compared to Ha-FAR-1 and Hf-FAR-1, the binding of Ha-FAR-2 is much weaker (Figs 5 and 6), perhaps due to the extended C-terminal fragment or different post-translational modification or perhaps do to its preference for structurally different ligands. Competition assays in this study showed oleic acid competitively displaced not only DAUDA but also retinol (Fig 7), thereby indicating that the two distinct binding pockets might be interactive. Similarly, previous reports indicated that Gp-FAR-1, Ab-FAR-1 and Ce-FAR-7 have distinct binding pockets for fatty acids and retinoids [15,27,31]. The result of in situ hybridization showed that the mRNAs encoding Ha-FAR-1 and Ha-FAR-2 were present in the hypodermis (Fig 8) and had hydrophobic secretory signal peptides. These results are consistent with investigations of other nematode FAR proteins. For example, the Gp-FAR-1 protein was detected on the surface of freshly hatched preparasitic J2 of *G*. *pallida* by immunolocalization studies and its mRNA was present in the hypodermis [27]. Similar results with hypodermal localization of *far* mRNA were obtained with *A*. *besseyi* FAR-1 [15,42]. The hypodermis is very active metabolically and plays an important roles in absorption of compounds from the external environment and in storage of metabolic reserves [43]. The excretory/secretory (ES) products of several mammal-parasitic nematodes are known to contains FAR proteins [44]; for example, the occurrence of Ac-FAR-1 in *A*. *caninum* \ES products and somatic extracts [26] indicates that secreted FAR protein exerts a role in host tissue. In *H*. *avenae*, *far* genes are transcribed and translated in the hypodermis and then released through the cuticle, as often occurs in many nematodes. In *M*. *hispanica*, the mRNA of FAR-1 was localized in the subventral esophageal glands and possible secretion into host tissue through the stylet [45] as are other phytoparasitic nematode secretory proteins.

The qRT-PCR results showed that the *Ha-far-1*and *Ha-far-2* were expressed in all developmental stages examined, indicating that both are essential for the entire nematode life cycle. The two *far* genes have discrete transcriptional patterns, with *Ha-far-1* particularly greater than *Ha-far-2* in all developmental stages (Fig 9). Curiously, the highest expression level of *Ha-far-1* was observed in J4, whereas for postparasitic J2 exhibited the highest expression level of *Ha-far-2*. The results demonstrate that the two genes perhaps possess different biological functions, with *Ha-far-1* playing a key role in J4 an important pre-reproduction *Ha-far-2* playing a key role in the postparasitic J2 establishing and maintaining infection. Similarly, members of the *far* family of genes are differentially expressed in different developmental stages of *C*. *elegans* [31]. In animal-parasitic nematodes, the *Hc-far-1* gene expression in *Haemonchus contortus* was higher in adults than in larvae[24]; in *A*. *ceylanicum*, Ac-far-1 mRNA expression was lowest in males [26]. In plant-parasitic nematodes, the highest level *far* transcription in *A*. *besseyi* occurred in females [15]. The *Mj-far-1*gene in *M*. *javanica* was highly expressed in the second-stage juveniles [17]. In conclusion, the expression pattern of FAR genes varies among species and developmental stages in accordance with their biological functions.

Our study is the first to reveal two distinct FAR proteins within the same plant-parasitic nematode species: Ha-FAR-1 and Ha-FAR-2 had 28 percent identity at the amino acid level. In contrast, only one FAR protein has been reported in other species of phytoparasitic nematodes, such as Mj-FAR-1 from *M*. *javanica*, Rs-FAR-1from *R*. *similis*, Gp-FAR-1 from *G*. *pallida* and Ab-FAR-1 from *A*. *besseyi* [15,25,27,42], and the *H*. *filipjevi* reported herein. Among other nematodes, *C*. *elegans* notably produces multiple isoforms of FAR proteins [31], and the animal parasite *A*. *caninum* produces two FAR orthologues (Ac-FAR-1 and Ac-FAR-2) suggested to be controlled by alleles. Sequence identity between Ac-FAR-1and Ac-FAR-2 is 91 percent at the amino acid level, and no significant difference was found in molecular mass [26,29]. In contrast, our discovered sequence identity (28%) and molecular mass differences between Ha-FAR-1 (17kDa) and Ha-FAR-2 (30kDa) are dramatic. Moreover, the two proteins were encoded by distinct genes; phylogenetic analysis indicated that *Ha*-FAR-1, *Ha*-FAR-2 and *Hf*-FAR-1were present in one group, *Ha*-FAR-2 was in a self-contained branch within the plant- and animal-parasitic nematode clusters (Fig 2). These results suggest a paralogous rather than an allelic relationship, a possibility that requires confirmation by genomic localization and evolutionary analysis. The theoretical pI of Ha-FAR-1 and Ha-FAR-2 was 5.62 and 9.02, respectively, indicating that the two proteins may play a role in different cellular environments. *H*. *avenae* and *H*. *filipjevi* are closely related species with very minor molecular and morphological differences [5,33,46], and sequence similarities illustrated by sequence alignment and phylogenetic analysis showed that identity between Ha-FAR-1 and Hf-FAR-1 was significantly high (96%), and grouped together in the phylogenetic tree (Fig 2), thereby indicating that the function, origin and evolution of the two genes were similar. Remarkably, no Hf-FAR-2 was detected in *H*. *filipjevi*, neither by searching its transcriptomic database (unpublished data) nor by PCR with the primers designed based on the cDNA sequence of *Ha-far-2*. It is possible that, like other plant-parasitic nematodes, *H*. *filipjevi* contains only a single FAR protein.

In conclusion, we made a preliminary attempt to characterize and understand *far* genes in cereal cyst nematodes. Our results provide the first indication that plant-parasitic nematodes may possess two FAR proteins originating from different genes and that both gene duplication and post-transcriptional modifications may be used to generate diverse FAR proteins.
