# Correction: Characterization of Three Novel Fatty Acid- and Retinoid-Binding Protein Genes (*Ha-far-1*, *Ha-far-2* and *Hf-far-1*) from the Cereal Cyst Nematodes *Heterodera avenae* and *H*. *filipjevi*

**DOI:** 10.1371/journal.pone.0165356

**Published:** 2016-10-20

**Authors:** The PLOS ONE Staff

Fig 9 appears incorrectly in the published article. Please see the corrected Fig 9 and its caption below. The publisher apologizes for the error.

**Fig 9:**
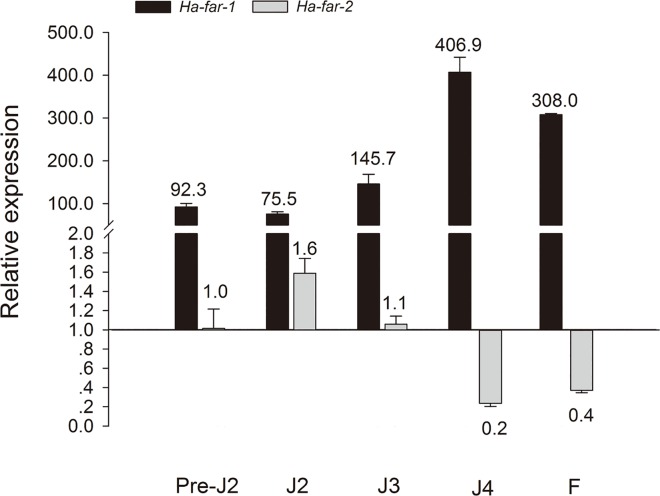
Relative expression of *Ha-far-1* and *Ha-far-2* in five developmental stages was determined using RT-qPCR. The relative expression was calculated with 2^-ΔΔCt^ method by normalizing with two internal reference β-actin gene and GAPDH gene and presented as the change in mRNA level in various nematode developmental stages relative to that of *Ha-far-2* at preJ2. preJ2: pre-parasitic second-stage juvenile; J2, J3 and J4: second-, third- and fourth-stage juvenile, respectively; F: female.
